# Acquired Hemophilia A: A Potentially Fatal Bleeding Disorder

**DOI:** 10.7759/cureus.8744

**Published:** 2020-06-21

**Authors:** Navdeep Singh, Sandeep Singh Lubana, Lech Dabrowski

**Affiliations:** 1 Hospice and Palliative Care Medicine, North Shore Long Island Jewish Hospital, Manhasset, USA; 2 Hematology and Medical Oncology, State University of New York Downstate Medical Center, Brooklyn, USA; 3 Hematology and Medical Oncology, Brookdale Hospital Medical Center, Brooklyn, USA

**Keywords:** hemophilia-a, autoimmune, immunosuppressive

## Abstract

Acquired hemophilia A (AHA) is a rare autoimmune hematological disorder that has an incidence of about 1.5 cases per million people per year. It occurs in the elderly with the median age of 75 years, and most of the cases are idiopathic. It occurs due to the development of factor VIII inhibitor, which is an autoantibody against factor VIII leading to potentially life-threatening bleeding episodes. The diagnosis of AHA is often delayed and challenging. We report a case of an 86-year-old male who initially presented with signs and symptoms of a stroke. He was found to have oral mucosal bleeding and swelling of the floor of the mouth. He later developed epistaxis, hematuria, and melena. He had an isolated elevation of activated partial thromboplastin time (APTT) with very high levels of factor VIII inhibitor (1152 Bethesda units) and very low levels of Factor VIII (<1%). He was managed with supportive transfusion, bypass agents, and immunosuppressive therapy. AHA is a rare autoimmune bleeding disorder and is more commonly seen in the elderly population. Bleeding in AHA is usually sudden and sometimes life-threatening. Hence early hemostasis with bypassing agents and treatment with immunosuppressive agents should be initiated. Due to the rarity of the disorder, it is crucial to report AHA cases to create awareness and increase the index of suspicion of the clinicians for early diagnosis and treatment to prevent morbidity and mortality.

## Introduction

Acquired hemophilia A (AHA) is a rare autoimmune hematological disorder in which patients present with spontaneous life-threatening bleeding without any personal or family history of bleeding disorders [1]. AHA has an incidence of about 1.5 cases per million people per year with the median age of 75 years, and most of the cases are idiopathic [2]. Patients with AHA can have mucocutaneous, soft tissue, gastrointestinal, or life-threatening intracranial bleeding [1,3]. The diagnosis of AHA is suspected in patients with an isolated elevation of activated partial thromboplastin time (APTT) and when mixing studies fail to correct APPT. Low factor VIII levels and high factor VIII inhibitor levels establish the diagnosis of AHA [1,4]. Two main strategies employed in the management of AHA are achieving hemostasis and immunosuppression [5-6].

We describe a case of a patient presenting with massive swelling of the floor of the mouth requiring urgent nasotracheal intubation to secure the airway. He had a prolonged hospital course complicated by epistaxis requiring bilateral nasal packing, gross hematuria, and melena with successful discharge.

## Case presentation

An 86-year-old man with a past medical history of hypertension was brought to the emergency room with concerns of stroke after his wife noticed slurred speech and dysphagia that worsened over the course of few hours. On physical examination, the patient had tense beefy red swelling of the floor of the mouth, and the oropharynx was unable to be visualized. The patient had bloody oral mucosal secretions. The neck was mildly swollen (right > left) and tender. There was no stridor noticed. There was no history of trauma. The patient did not have any history of smoking, alcohol, or illicit drug use. He did not have any personal or family history of cancer or bleeding disorders. He was not on aspirin or anticoagulation.

Fiberoptic examination revealed significant swelling and beefy red coloration of the base of the tongue, epiglottis, and right lateral pharyngeal walls. The nasopharynx and oropharynx were normal. Due to the acute nature of the swelling and unclear etiology, the patient had fiberoptic nasotracheal intubation to secure the airway.

**Figure 1:**
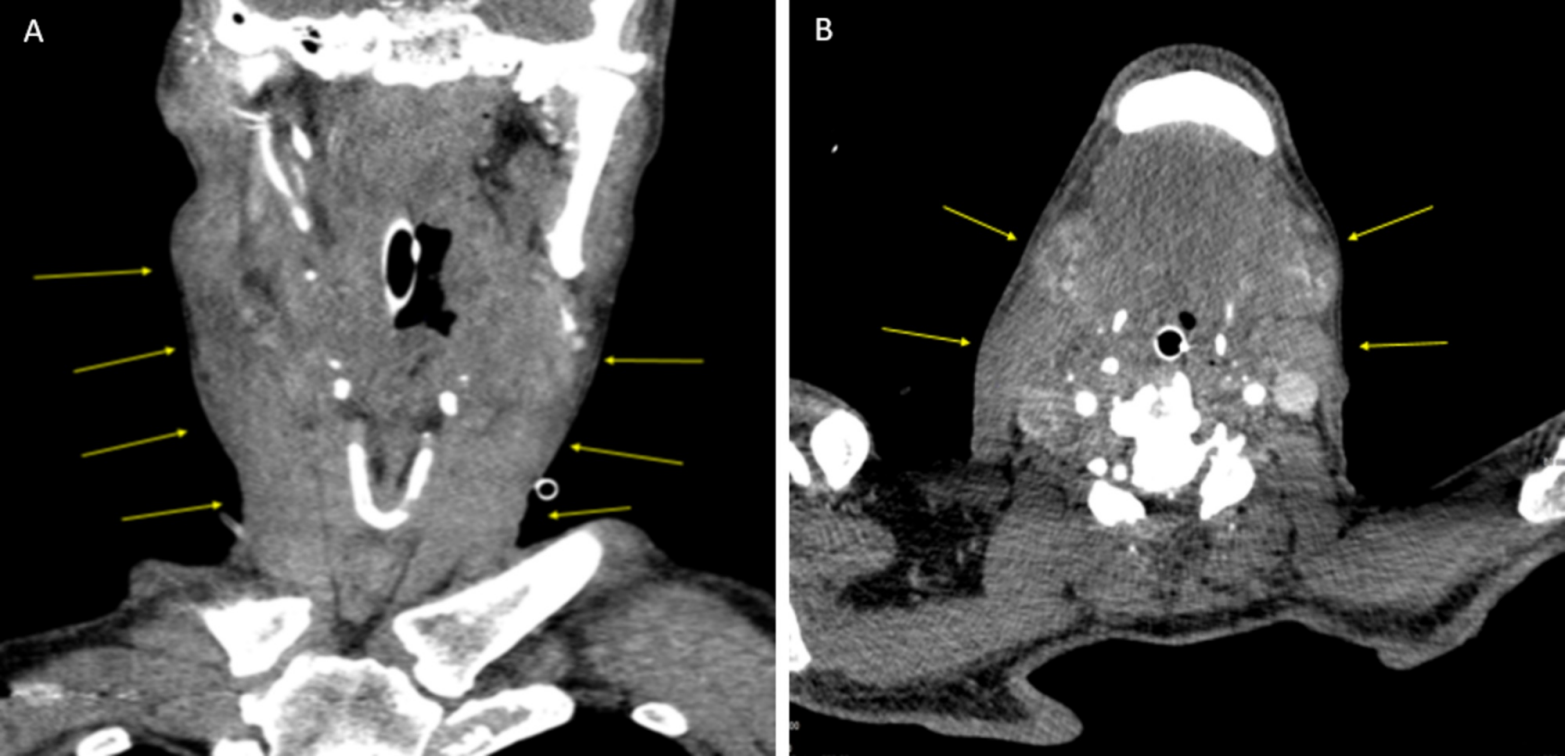
Computerized tomography of neck (A) Coronal section and (B) Transverse section: revealing diffuse subcutaneous edema (yellow arrows indicating edema) of the sublingual and submandibular regions.

On initial laboratory investigation (lab), hemoglobin was 9.3 mg/dl. However, it dropped to 6.4 mg/dl the next day due to ongoing blood loss. The patient’s platelet count was 344 x 10^9^/L. He had transfused three units of packed red blood cells (PRBC) and two fresh frozen plasma (FFP). His initial activated partial thromboplastin time (aPTT) was elevated at 53.2 seconds (reference range 25-35 seconds) with normal prothrombin time and normal international normalized ratio (INR). Mixing studies failed to correct aPTT indicating the presence of inhibitors. Factor VIII (FVIII) assay was <1% (reference range 50-150 IU/dL). Further testing revealed an extremely high factor VIII inhibitor level of 1152 Bethesda units (reference range </= 0.50). As mixing study did not correct aPTT (41.0 sec-immediate mix; reference range 22.0-29.0 sec) and the patient had low level of factor VIII along with high inhibitor titers, this led to the diagnosis of acquired hemophilia. Liver function tests were within normal limits. Prostate-specific antigen (PSA) level was 0.47 ng/mL (reference range 0.0-4.0 ng/mL). A recent colonoscopy was negative for any malignancy. Further malignancy workup included computerized tomography (CT) of chest, abdomen, and pelvis which was negative. Infectious workup for hepatitis B and C along with rheumatologic serological workup was negative for a secondary cause of AHA. CT of the neck revealed diffused subcutaneous edema of the sublingual and submandibular regions (Figure 1). No discrete hematoma or active extravasation of intravenous contrast was noted.

He was started on prednisone 1 mg/kg daily and bypassing agent, the activated prothrombin complex concentrate (APCC) at 70 units/kg every 8 hours. He required transfusion support with multiple PRBCs, FFPs, and cryoprecipitates. The oral mucosal bleeding and swelling of the oral cavity improved, and the patient was extubated on day 8 of the hospitalization.

However, following extubation the patient started to have right nasal cavity bleeding. Multiple clots were suctioned out from the nasal cavity and posterior oropharynx. Nasal bleeding failed to stop with oxymetazoline sprays and manual pressure; hence right nasal packing was done on day 9. The next day, the patient started oozing from left naris, which required the use of surgiflo with thrombin and then left nasal packing. The patient also developed gross hematuria with clots for which bladder irrigation and aspiration was done. Due to the continued bleeding rituximab 375 mg/m^2^ was initiated on day 11 with a plan to add oral cyclophosphamide when the patient can swallow. The prednisone was switched to methylprednisone 60 mg daily.

On day 15, nasal packing was removed with improvement in nasal bleeding. Throughout the course of the hospitalization, the patient continued to have intermittent oral and nasal mucosal bleeding and hematuria for which he continued to require APCC. On day 29, the patient was given cycle 3 of rituximab and intravenous cyclophosphamide at 2 mg/kg/day was added as the patient continued to bleed. The patient received cyclophosphamide for five days, and additional doses were held as the patient started to improve clinically. Rituximab was continued for a total of four weekly doses.

Factor VIII levels started to increase, and inhibitor titer down trended following administration of immunosuppressive therapy (Table 1). aPTT normalized following the administration of steroids, rituximab, and cyclophosphamide. He continued to improve clinically and did not require any bypassing agents over the last few days. His hemoglobin became stable at around 9-10 gm/dl. On day 45, the patient was discharged with prednisone at 60 mg PO daily with a tapering dose.

**Table 1:** Initial lab values upon presentation aPTT: activated partial thromboplastin time; PT: prothrombin time; INR: international normalized ratio; BUN: blood urea nitrogen.

	Admission	Following cycle 1 of Rituximab	Following cycle 2 of Rituximab	Following cycle 3 of Rituximab and 5 doses of cyclophosphamide	Following cycle 4 of Rituximab (On discharge)	Reference range
Factor VIII assay (%)	<1	<1	3	5	38.6	50-150
Factor VIII inhibitor (BU/ml)	1152	960	NP	768	288	Negative
aPTT (seconds)	53.2	35	33	31	29.7	28-38
PT (seconds)	10.2	11.8	11.7	11.6	11.4	8.5-11.5
INR	1	1.2	1.1	1.1	1.1	0.9-1.2
White blood cells (10^3^/uL)	7.96	20	13	14.42	9.58	3.60-9.50
Hemoglobin (g/dl)	9.3	9.4	7.4	8.0	9.2	13-17
Hematocrit (%)	31.1	30.7	23.4	25.8	29.0	39-49
Platelets (10^3^/uL)	308	431	207	225	224	150-440
BUN (mg/dl)	8	12	20	15	11	7-20
Creatinine (mg/dl)	0.90	0.70	0.64	0.80	.062	0.6-1.2

On follow-up the patient was found to have elevated aPTT of 35.5 seconds with normal PT and elevated Factor VIII inhibitor level of 144 BU. In order to prevent future bleeding episodes and to normalize the inhibitor levels cyclophosphamide 100 mg PO daily was added to prednisone 60 mg daily. After four months of outpatient follow-up laboratory investigation revealed a normal aPTT, PT, factor VIII assay of 41, and factor VIII inhibitor of 5.5 BU.

## Discussion

AHA, also known as acquired factor VIII inhibitor disorder, is caused by the inhibitory autoantibodies that inactivate factor VIII [7]. It is a rare disorder with a reported annual incidence of 1.5 cases per million per year. It is most frequently seen in the elderly population, and the median age of diagnosis is 75 years [2]. Males and females are equally affected; however, more cases are reported in women of childbearing age (20-40 years) related to pregnancy [8-9]. Most of the cases are idiopathic; however, several risk factors are implicated in AHA, the most common being pregnancy, post-partum status, and autoimmune disorders. The other risk factors include malignancies, dermatological disorders, and medications [7,10]. In our case, the patient did not have any underlying medical conditions and was not on any medication which could be related to AHA. Physical examination and laboratory investigations also did not determine a cause for his AHA.

Lozner et al. in 1940 described the first case of AHA, in an elderly male who suffered from bleeding diathesis after undergoing surgery [11]. The acquired and the congenital AHA differ in the severity and the way they present. The acquired AHA is more severe and presents with skin, soft-tissues, and mucosal bleeding. Hemarthrosis is uncommon in AHA. Compared to congenital hemophilia A, AHA is associated with a high mortality rate, which is secondary to either retroperitoneal or intracranial bleeding. In congenital AHA, bleeding is mainly in the joints [1,3]. Our patient presented with oral and nasal mucosal bleeding and later developed hematuria.

Diagnosis of AHA should be suspected in patients who present with new, unexplained bleeding without a personal and family history of the bleeding disorder. While the majority of patients present with mild to life-threatening bleeding, some of the patients are asymptomatic, with only prolonged aPTT on routine blood workup [1]. The diagnosis can be suspected in the context of an isolated prolonged aPTT that does not correct after 1:1 mixing study [4]. Mixing tests mix the patient’s plasma with that of a pooled normal plasma in a ratio of 1:1. The next crucial diagnostic step is the quantitative measurement of coagulation factors activity and coagulation factors inhibitor level. The FVIII inhibitor level is measured by the Bethesda assay and expressed as Bethesda units (BU) [3]. Low factor VIII activity and the presence of FVIII inhibitor confirms the diagnosis of AHA. However, the levels of either one do not correlate with the disease activity or severity of bleeding [1]. Also, aPTT levels do not have any correlation with the severity of bleeding. In a study of 501 patients, most of the patients (89%) were diagnosed with AHA after the bleeding event led to further investigations. The remaining patients did not have bleeding episodes at the time of the diagnosis. The diagnosis was made based on prolonged aPTT [12]. Our patient at the time of admission had a hemoglobin level of less than 7 g/dL. Further investigation revealed elevated aPTT and mixing studies failed to correct aPTT. Factor VIII activity was low, and inhibitor levels were significantly elevated.

Management of patients with AHA involves controlling the acute bleeding, preventing further bleeding, eradication of inhibitor, and termination of the underlying autoimmune process. The first and foremost step in the management of the AHA is to control acute bleeding and prevent further bleeding. This should be accompanied by the eradication of the coagulation factors inhibitors as the risk of bleeding remains high in the presence of inhibitor. The invasive procedures, including venous puncture, should be limited, as these can provoke or worsen the ongoing bleeding [5-6].

In patients with low factor VIII inhibitor titers (<5 BU) replacement with human factor VIII concentrates± DDAVP administration is recommended [3-13]. If the patient’s antibody titer is high (>5 BU), replacement with even high doses of human factor VIII concentrates, is ineffective, as the inhibitor will immediately block all the factor VIII administered, therefore these patients are managed with bypassing agents [9]. Bypassing agents consist of activated prothrombin complex concentrates (APCC) and recombinant factor VIIa (rFVIIa) with an eﬃcacy of 86% and 95%, respectively [3].

The usual recommended dose and frequency for FEIBA is 75 IU/kg/dose every 8-12 hours (maximum dose 200 IU/kg/day) and for rFVIIa (Novoseven) 90 μg/kg/dose every 2-3 hours. The frequency of the dosing also depends on the clinical response. Either one of them can be used depending on the response [14]. Recombinant porcine factor VIII (rpFVIII) is also another bypassing agent option. The initial recommended dose of rpFVIII is 200 U/kg and subsequent doses should be given to maintain Factor VIII Coagulant Activity (FVIII:C) trough levels >50% [15]. We used APCC for the management of bleeding in our patient.

The process of hemostasis should be accompanied by the eradication of the inhibitor once the diagnosis of AHA is confirmed and should be done without delay. Corticosteroids are the first line of therapy (prednisone, 1 mg/kg/day for 4-6 weeks), either alone or in combination with cyclophosphamide (1-2 mg/kg/day orally for a maximum of six weeks). Rituximab (375 mg/m^2^ weekly for four weeks) is used when the cyclophosphamide is contraindicated, or treatment failure occurs. It can be used alone or in combination with other agents [1]. Our patient had an inadequate clinical response to steroids alone, so a combination of steroids, rituximab, and cyclophosphamide was used, and adequate hemostasis was achieved with further bleeding episodes. Other alternatives that can be used include azathioprine, vincristine, mycophenolate, and cyclosporine [14]. The response to the inhibitor eradication therapy should be monitored by weekly/biweekly CBC, aPTT, FVIII activity, and inhibitor titers. After the completion of the treatment, the labs should be repeated every 2-3 months during the first year as the median time of relapse is reported to be 7-9 months [16]. Emicizumab, a bispecific recombinant monoclonal antibody, has been approved for prophylactic treatment of AHA to prevent further bleeding episodes. It replaces the function of missing activated factor VIII by bridging activated coagulation factor IX and to factor X, leading to activation of Factor X to achieve effective hemostasis [17].

## Conclusions

AHA is a rare life-threatening bleeding disorder. Clinicians should have high index of suspicion of AHA in an elderly patient presenting with sudden spontaneous mucocutaneous bleeding in the setting of an isolated elevated PTT and with no prior history of bleeding or anticoagulation use. Prompt treatment based on the initial suspicion can prevent devastating complications as AHA carries the risk of significant morbidity and mortality. Rapid achievement of hemostasis with bypassing agents is an important initial step in the treatment of AHA. The inhibitors should be eradicated with the use of immunosuppressive agents to prevent rebleeding episodes. Due to the rarity and acute nature of the disease the clinical trials are difficult to conduct, however better hemostatic and immunosuppressive agents are needed to develop for the better management of the disease.

## References

[1] Huth-Kuhne A, Baudo F, Collins P (2009). International recommendations on the diagnosis and treatment of patients with acquired hemophilia A. Haematologica.

[2] Franchini M, Mannucci PM (2013). Acquired hemophilia A: a 2013 update. Thromb Haemost.

[3] Elezovic I (2010). Acquired haemophilia syndrome: pathophysiology and therapy. Srpski arhiv za celokupno lekarstvo.

[4] Franchini M, Lippi G (2008). Acquired factor VIII inhibitors. Blood.

[5] Collins P, Baudo F, Huth-Kuhne A (2010). Consensus recommendations for the diagnosis and treatment of acquired hemophilia A. BMC Res Notes.

[6] Collins PW, Chalmers E, Hart D (2013). Diagnosis and management of acquired coagulation inhibitors: a guideline from UKHCDO. Br J Haematol.

[7] Cugno M, Gualtierotti R, Tedeschi A, Meroni PL (2014). Autoantibodies to coagulation factors: from pathophysiology to diagnosis and therapy. Autoimmun Rev.

[8] Kruse-Jarres R, Kempton CL, Baudo F (2017). Acquired hemophilia A: updated review of evidence and treatment guidance. Am J Hematol.

[9] Knöbl P (2018). Prevention and management of bleeding episodes in patients with acquired hemophilia A. Drugs.

[10] Kaur K, Kalla A (2018). A case of acquired hemophilia A in an elderly female. J Community Hosp Intern Med Perspect.

[11] Lozner EL, Jolliffe LS, Taylor FHL (1940). Hemorrhagic diathesis with prolonged coagulation time associated with a circulating anticoagulant. Am J Med Sci.

[12] Knoebl P, Marco P, Baudo F (2012). Demographic and clinical data in acquired hemophilia A: results from the European Acquired Haemophilia Registry (EACH2). J Thromb Haemost.

[13] Shetty S, Bhave M, Ghosh K (2011). Acquired hemophilia A: diagnosis, aetiology, clinical spectrum and treatment options. Autoimmun Rev.

[14] Charlebois J, Rivard GÉ, St-Louis J (2018). Management of acquired hemophilia A: review of current evidence. Transfus Apher Sci.

[15] Huth-Kühne A, Baudo F, Collins P (2009). International recommendations on the diagnosis and treatment of patients with acquired hemophilia A. Haematologica.

[16] Sborov DW, Rodgers GM (2013). How I manage patients with acquired haemophilia A. Br J Haematol.

[17] Franchini M, Marano G, Pati I (2019). Emicizumab for the treatment of haemophilia A: a narrative review. Blood Transfus.

